# Solid cryogen: a cooling system for future MgB_2_ MRI magnet

**DOI:** 10.1038/srep43444

**Published:** 2017-03-02

**Authors:** Dipak Patel, Md Shahriar Al Hossain, Wenbin Qiu, Hyunseock Jie, Yusuke Yamauchi, Minoru Maeda, Mike Tomsic, Seyong Choi, Jung Ho Kim

**Affiliations:** 1 Institute for Superconducting and Electronic Materials, Australian Institute for Innovative Materials, University of Wollongong, Squires Way, Innovation Campus, North Wollongong, New South Wales 2500, Australia; 2 Department of Physics, College of Science and Technology, Nihon University, Tokyo 101-8308, Japan; 3 Hyper Tech Research, Inc., 539 Industrial Mile Road, Columbus, Oh 43228, USA; 4 Busan Center, Korea Basic Science Institute, Busan 609-735, Republic of Korea

## Abstract

An efficient cooling system and the superconducting magnet are essential components of magnetic resonance imaging (MRI) technology. Herein, we report a solid nitrogen (SN_2_) cooling system as a valuable cryogenic feature, which is targeted for easy usability and stable operation under unreliable power source conditions, in conjunction with a magnesium diboride (MgB_2_) superconducting magnet. The rationally designed MgB_2_/SN_2_ cooling system was first considered by conducting a finite element analysis simulation, and then a demonstrator coil was empirically tested under the same conditions. In the SN_2_ cooling system design, a wide temperature distribution on the SN_2_ chamber was observed due to the low thermal conductivity of the stainless steel components. To overcome this temperature distribution, a copper flange was introduced to enhance the temperature uniformity of the SN_2_ chamber. In the coil testing, an operating current as high as 200 A was applied at 28 K (below the critical current) without any operating or thermal issues. This work was performed to further the development of SN_2_ cooled MgB_2_ superconducting coils for MRI applications.

Magnetic resonance imaging (MRI) represents one of the greatest achievements among modern medical technologies. It is a non-invasive monitoring and diagnostic tool that is used to collect images of the inside of the human body, which can aid in the diagnosis of several health conditions. To produce highly detailed images in an MRI, magnetic fields are required that are both strong and homogeneous. In most of the currently installed MRI systems, niobium-titanium (Nb-Ti) superconducting magnets have been widely used^1^,^2^. The Nb-Ti based magnets, however, need to be cooled down to and operated at 4.2 K in an expensive liquid helium (LHe) bath due to Nb-Ti’s low critical temperature (*T*_c_) of 9.8 K^3^. Anticipation of increasing LHe prices and possible shortages in the near future have increased demand for LHe-free MRI magnets^1^,^3^. To fulfill the above requirements, magnesium diboride (MgB_2_) has great potential among the conventional commercialized low- and high-temperature superconductors^4^,^5^,^6^,^7^,^8^,^9^,^10^,^11^,^12^,^13^,^14^,^15^,^16^,^17^,^18^. Importantly, the higher *T*_c_ (39 K) of MgB_2_ offers a larger thermal margin than the 9.8 K of Nb-Ti, even under LHe operation^19^,^20^,^21^.

When considering LHe-free environments, the cost and weight of coolant are vital parameters to inform the operational conditions of the superconducting magnet. From this point of view, an inexpensive and lightweight solid nitrogen (SN_2_) system could be the best choice due to its high heat capacity when combined with a cryocooler^18^,^22^,^23^,^24^,^25^,^26^. In particular, there are three major benefits to the use of SN_2_ as a next-generation cryogen for the MgB_2_ superconducting magnet: (i) thermal stability, (ii) ease of operation, and (iii) capability of operating with an unreliable-power-source^27^.

The first cooling system employing SN_2_ cryogen was proposed, designed, and tested with six double-pancake coils made of Bi_2_Sr_2_Ca_2_Cu_3_O_*x*_ (BSCCO-2223)/Ag tape by Haid *et al*.^27^. Soon afterwards, Song *et al*. investigated the SN_2_ cooling system for a high temperature superconducting (HTS) fault current limiter^28^. Bascuñán *et al*. also reported a niobium-tin (Nb_3_-Sn) superconductor based magnet, cooled down to 4.2 K using hybrid cryogen cooling, such as with SN_2_ and LHe^29^. Recently, Yao *et al*. reported the test results for MgB_2_ solenoid coils utilizing SN_2_ as a cryogen for the first time^18^. Their assembled magnet was prematurely quenched, however, at currents ranging from 79 A to 88 A, even though each coil had a current carrying capacity of over 100 A. For better thermal stability, Kim *et al*. designed and evaluated the performance of a hybrid cryogen cooling system using both SN_2_ and liquid neon for HTS magnetic energy storage^30^. Most recently, we reported persistent-mode operation of an MgB_2_ magnet at ~20 K in SN_2_, with the magnet carrying 100 A for 4.75 days, and successfully explored the possibilities of SN_2_ as a cryogen^31^.

For cryostat fabrication, materials for the cryogen reservoir, such as stainless steel (SS), copper (Cu), and aluminium (Al), are often used because of their suitable structural and thermal properties in a cryogenic environment. It has been reported, however, that, due to the different thermal expansion coefficients when dissimilar materials are joined, particularly for joints between SS and Cu, leaks can develop at cryogenic temperatures in a cooling system utilizing cryogens^32^. For this reason, it is highly desirable to avoid dissimilar material joints in cooling systems where cryogens and high vacuum are involved. In fact, Cu is known to provide high temperature uniformity in conduction cooling systems due to its high thermal conductivity^26^. Nevertheless, it can deliver a high conductive heat load when it is used in the cryogenic apparatus to connect room temperature (RT) to low-temperature structures. Therefore, SS is often chosen as the structural material in cryostats due to its low thermal conductivity and high yield strength. For this reason, in our designed and fabricated SN_2_ cooling system, SS304L steel was specified as the structural material, except for the radiation shield, to avoid dissimilar material joints. To fulfil this requirement, however, detailed thermal design for the SN_2_ cooling system was required.

In this work, therefore, we firstly obtained numerical results through a finite element analysis (FEA) simulation to explore the feasibility of the design for the SN_2_ cooling system. From the FEA results, we concluded that, as a result of the low thermal conductivity of the SS, a temperature gradient would be generated across the SN_2_ chamber, even though the total heat load on the SN_2_ chamber was lower than the cooling capacity margin. Based on these designs, we report a novel approach to enhance temperature uniformity in a conduction-cooled SN_2_ chamber. We also report on the design, fabrication, and testing of the SN_2_ cooling system.

## Design

### Thermal Design

The most important issue in designing a cooling system is a precise estimation of the total heat load on it in advance. In particular, the heat loads at various positions on the radiation shield and SN_2_ chamber, which includes conduction, radiation, residual gas conduction, and joule heating, need to be precisely evaluated. In detail, the conduction, radiation, and joule heating were calculated based on standard analytical models, whereas empirical values were used for the residual gas conduction, based on previous reports^26^,^33^. The access tubes from RT to the SN_2_ chamber were brazed at the radiation shield to mechanically secure them. Even though a thin-wall tube would be better for reducing conduction heat flow, thick-wall tubes were required to protect against mechanical deformation during the brazing process. To minimize this conduction heat flow, in this design, at an optimized location, the access tube thickness was reduced to 0.5 mm for 20 mm length. Details of the estimation methodology for various components of the total heat loads are presented in the Supporting Information. A summary of the estimated heat loads on the SN_2_ cooling system is listed in Table 1. The total estimated heat load on the radiation shield was 18.61 W and 45.10 W, without current and with 200 A current, respectively. On the other hand, the total estimated heat load on the SN_2_ chamber was 0.46 W and 0.72 W, respectively, under the same operating conditions. The steady-state heat loads without electric current injection were well matched with the available cooling power of 40 W at 43 K at the 1^st^ stage and 1 W at 4.2 K at the 2^nd^ stage of the cryocooler.

### Mechanical Design

The mechanical design of the SN_2_ cooling system was carried out as per the 2007 American Society of Mechanical Engineers (ASME) boiler and pressure vessel code viii, division 2^34^, and the 2007 ASME boiler and pressure vessel code ii, part D: Properties of materials^35^. Cu, with its higher thermal conductivity was not practical for fabricating the SN_2_ chamber due to the need to avoid joints between dissimilar materials. Therefore, it was necessary to use SS, even though it had the drawback of lower thermal conductivity^26^. The SN_2_ chamber and the cryostat were designed for an internal 0.3 MPa gauge pressure, as under operation conditions, the pressure inside the SN_2_ chamber would be approximately 0.1 to 0.15 MPa (during liquid nitrogen (LN_2_) transfer and gas boil-off). The cryostat was designed for an internal maximum gauge pressure of up to 0.3 MPa to protect against any possible accidental leak in the system.

### Finite Element Analysis

The structural integrity of the SN_2_ cooling system was verified to be below the allowable specification of mechanical strength of the components to avoid failure if the chamber experienced cyclic repetition of the highest possible thermal expansion during the cool-down and warm-up processes. To simulate the thermal and mechanical design of the SN_2_ cooling system, the stationary FEA simulation of the entire cooling system were conducted in three parts: (i) the SN_2_ chamber with access tubes up to the radiation shield and the Cu rod; (ii) the radiation shield with tubes up to the top flange of the cryostat; and (iii) the cryostat with tubes up to the SN_2_ chamber. In each simulation, the temperature distribution, von Mises stress, and total displacement due to the loading conditions were estimated, except for the temperature distribution in the cryostat, since it remained at RT. A summary of the mechanical design parameters and FEA simulation results is presented in Table 2. The maximum von Mises stress and total displacement under self, magnet, and SN_2_ weight were estimated to be 9.17 MPa and 0.004 mm, respectively, on the SN_2_ chamber. The allowable stress in SS304 is up to 138 MPa^36^, as such, 9.17 MPa was deemed an acceptable value, and 0.004 mm was negligible displacement. Likewise, the other design parameters were also well within the allowable limits^26^,^36^.

**Figure 1:**
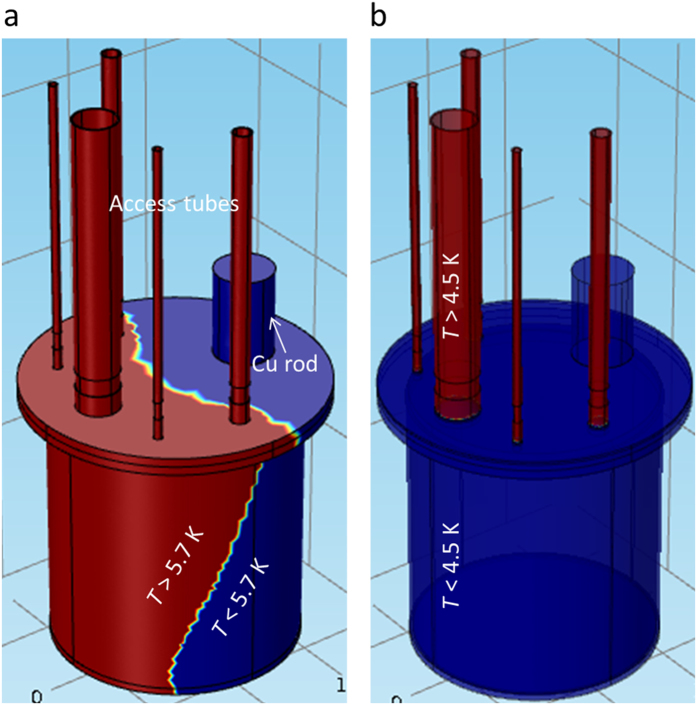
Temperature distribution in the SN_2_ chamber: (**a**) without Cu flange installation, and (**b**) with Cu flange installation on the SN_2_ chamber.

**Figure 2:**
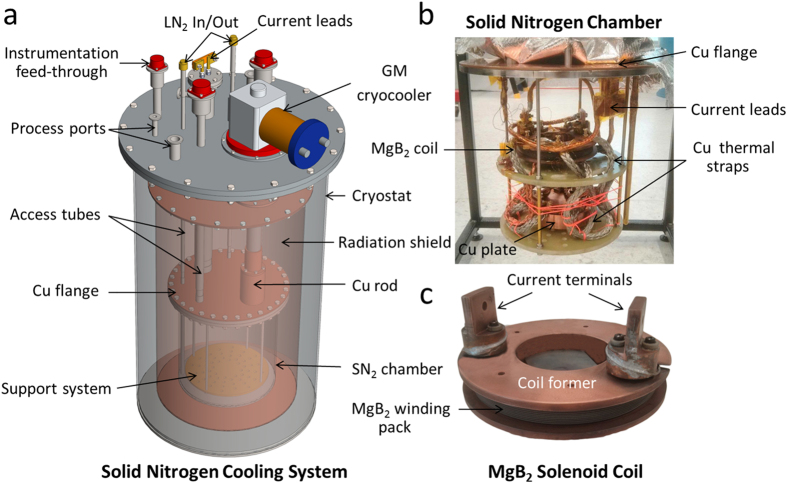
(**a**) 3D model of the designed and fabricated SN_2_ cooling system, (**b**) SN_2_ chamber, (**c**) digital image of the fabricated MgB_2_ solenoid coil.

To further examine the temperature distribution on the SN_2_ chamber, an FEA simulation of the SN_2_ chamber with the access tubes up to the radiation shield and Cu rod was performed. Figure 1a shows the temperature distribution on the designed SN_2_ chamber (see Fig. 2a). It can be seen there is a noticeable temperature gradient on the SN_2_ chamber, even though the total heat load on the SN_2_ chamber was well below the cooling margin. This clearly indicates that the 2^nd^ stage of the cryocooler was unable to extract the conductive heat load (using 1 W cooling power at 4.2 K) from the radial direction due to the lower thermal diffusivity of the SS^26^. To mitigate this temperature gradient, we developed a novel approach, in which a temperature moderator consisting of a Cu flange 3 mm in thickness was installed on the SN_2_ chamber. Figure 1b shows the temperature distribution on the SN_2_ chamber after installation of the Cu flange. As can be seen, the installation of the temperature moderator effectively removes the heat load on the 2^nd^ stage of the cryocooler, resulting in a uniform temperature on the SN_2_ chamber. Finally, based on this optimized design, the SN_2_ cooling system was fabricated.

### Installation

Figure 2a and b show the three-dimensional (3D) configurations of the SN_2_ cooling system and the SN_2_ chamber inside, respectively. As can be seen, the SN_2_ cooling system mainly consists of the cryostat, the radiation shield, the SN_2_ chamber, and the cryocooler. Importantly, the Cu flange was also installed on the top of the SN_2_ chamber (i.e. the SS flange). For better thermal contact between the SS and Cu flanges, Apiezon^®^ N grease was applied between two flanges^37^. The SN_2_ chamber was leak-tightened using 2 mm diameter indium wire between the two flanges^38^. The cryostat, SN_2_ chamber, and all access tubes were made of SS304L, whereas the radiation shield and flange were made of oxygen-free high-conductivity copper. The whole SN_2_ cooling system was cooled using a Sumitomo (model RDK-408E2 Gifford-McMahon (GM)) two-stage cryocooler, having the cooling capacity of 40 W at 43 K at the 1^st^ stage and 1 W at 4.2 K at the 2^nd^ stage. The 1^st^ and the 2^nd^ stages were thermally connected with the radiation shield and the SN_2_ chamber, respectively. Multilayer insulation (10 layers) was wrapped around the SN_2_ chamber and the radiation shield to minimize the radiation heat load. Vacuum of ≤2 × 10^−6^ torr was maintained throughout operation to minimize the residual gas conduction heat load^26^. For energizing the magnet, hybrid current leads were fabricated using brass and HTS tape^26^.

To validate the SN_2_ cooling system, an MgB_2_ solenoid coil (Fig. 2c) was fabricated using the ‘wind and react’ method. Heat treatment of the coil was carried out at 675 °C for 60 min under flowing argon gas. The specifications of the solenoid coil are listed in Table 3. This coil was then installed in the SN_2_ chamber, as shown in Fig. 2b. The Cu thermal straps were used to minimize the temperature gradient across the coil during cooling from 300 K to 77 K. The inductance of the coil was first evaluated using an FEA simulation, which was validated by using an induced inductive voltage during charging of the coil. The field constant of the solenoid was simultaneously calculated using the FEA simulation and verified by the standard solenoid magnetic field formula^39^. The critical current (*I*_c_) of the coil was characterized by using the criterion of 1 μV · cm^−1^. The *T*_c_ of the coil was measured by applying a 10 mA constant current from 37.6 K until the superconducting transition occurred.

**Figure 3:**
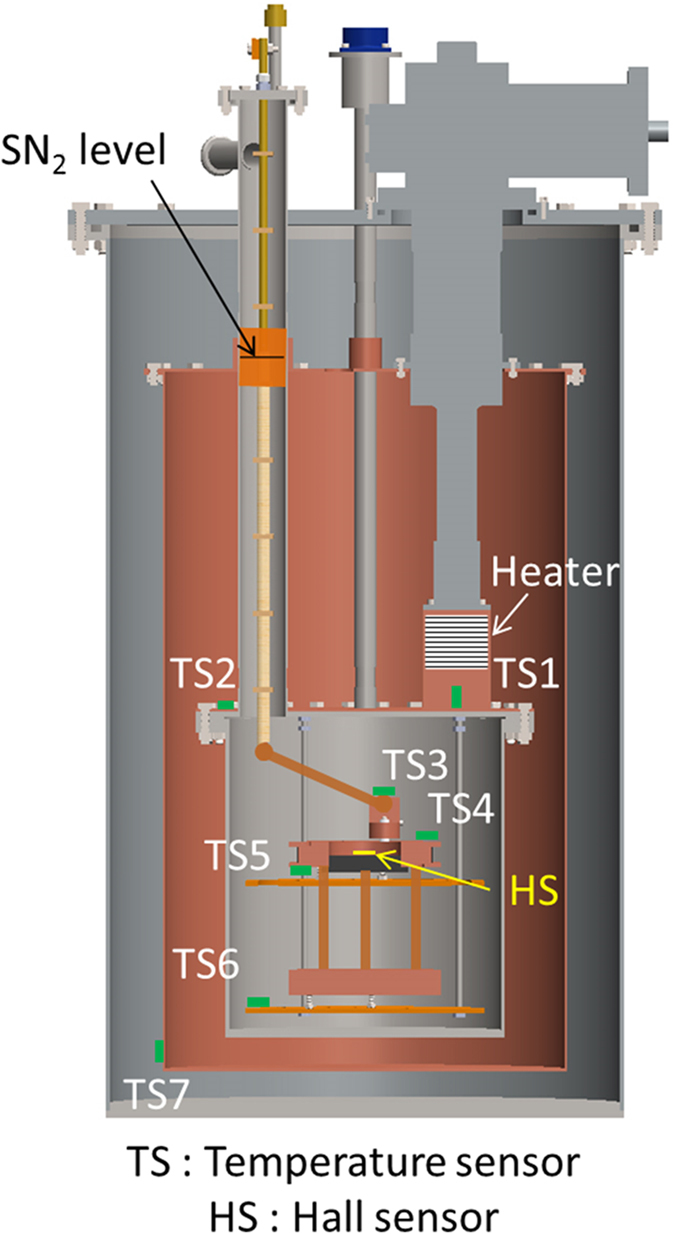
Cross-sectional view of the SN_2_ cooling system, including a schematic illustration of the temperature sensors, Hall sensor, and heater. The level of SN_2_ was up to the radiation shield flange, as shown in the figure.

As shown in Fig. 3, seven cryogenic temperature sensors were installed to monitor the temperature at different locations, denoted as TS1 to TS7^40^. A 50 Ω nichrome (32 AWG) heater was installed on the Cu rod below the 2^nd^ stage of the cryocooler to control the SN_2_ chamber temperature^41^. The heater was controlled using a temperature controller (Cryocon 32B)^42^. At the center of the coil, a Hall sensor (0.1 G sensitivity) was installed to measure the magnetic field generated by the coil. Flexible Cu leads were used for connections between the coil current terminals and the current leads.

## Results

**Figure 4:**
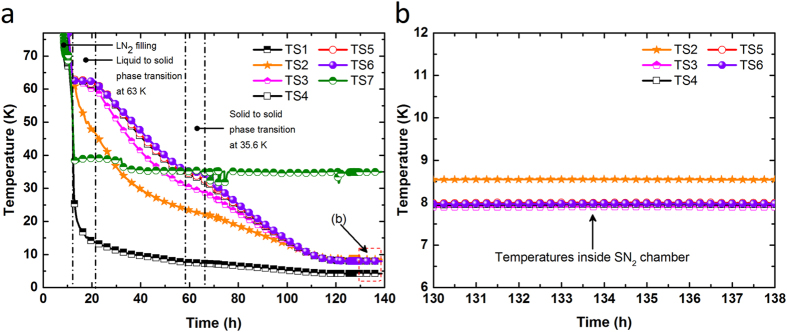
Temperature versus time curves of the cooling system: (**a**) from 77 K and (**b**) after 130 h of the cool-down. Temperature sensor locations: TS1 (on the Cu bar below the 2^nd^ stage of the cryocooler), TS2 (on the Cu plate of the SN_2_ chamber near the current lead tube), TS3 (on the negative current lead termination of the coil), TS4 (on top of the coil), TS5 (on the bottom of the coil), TS6 (on the G10 support plate), TS7 (on the radiation shield bottom, diagonal to the cryocooler 1^st^ stage connection).

Two important ways to confirm the capability of the SN_2_ cooling system were the temperature cooling profile and the MgB_2_ solenoid coil tests. A temperature profile at different positions inside the SN_2_ cooling system during cooling down is shown in Fig. 4a. It should be noted that the typical two-phase transitions for LN_2_ were observed during the cool-down process: the first is from liquid to solid at 63 K (~9.8 h) and the other is from solid to solid at 35.6 K (~7.9 h)^28^. Eventually, the inside of the SN_2_ chamber was cooled down to a consistent 8 K after approximately 130 h (Fig. 4b). It was observed that the SN_2_ level reached up to the top flange of the radiation shield inside the SN_2_ chamber (see Fig. 3). Thus, the SN_2_ itself was delivering an additional conductive heat load from the radiation shield to the SN_2_ chamber. This prevented the SN_2_ chamber from reaching any lower temperatures. Once the whole SN_2_ chamber temperature dropped below 10 K, there was no temperature difference between the MgB_2_ coil top (TS4) and its bottom (TS5). Eventually, the temperature in the entire SN_2_ chamber remained constant at around 8 K, indicating that the strategic Cu flange installation on the top of SN_2_ chamber could establish a uniform temperature distribution, as was anticipated.

**Figure 5:**
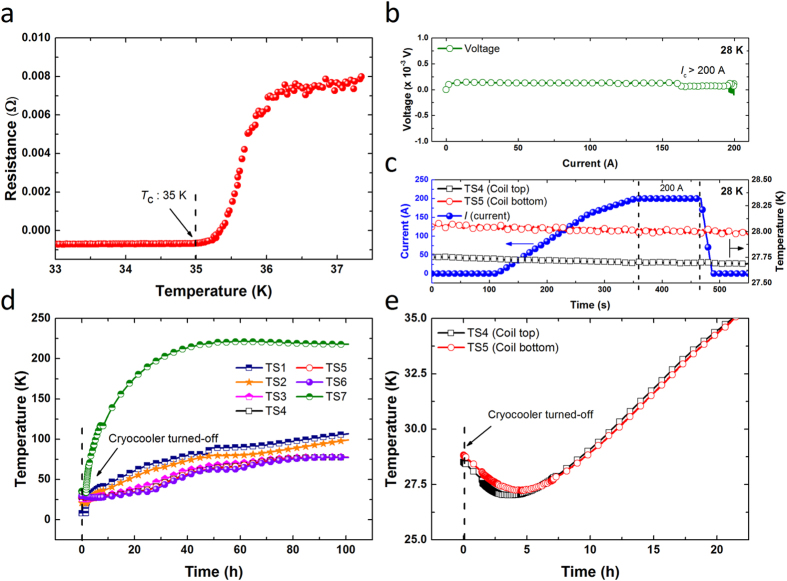
(**a**) Resistance versus temperature curve, (**b**) voltage versus current curve (at 28 K), (**c**) current and temperature versus time curves (at 28 K) of the solenoid MgB_2_ coil, (**d**) temperature versus time curves of the cooling system, and (**e**) temperature versus time curves of the temperatures on the coil after turning-off the cryocooler at ~28 K.

Before the solenoid coil test in the SN_2_ chamber, we characterized the *T*_c_ (Fig. 5a) and field constant of our MgB_2_ solenoid coil. The resistance of the solenoid coil was measured at a 10 mA constant current while decreasing the operating temperature. The *T*_c_ was estimated to be 35 K. To measure the field constant of the coil, the coil was energized up to 10 A current at 31.5 K. The measured and calculated field constant of the solenoid coil was 2.22 G · A^−1^ and 2.19 G · A^−1^, respectively^39^. Figure 5b shows the *I*_c_ measurement results (>200 A) of the solenoid coil at 28 K, which is close to the *T*_c_, to confirm the thermal stability of the SN_2_ cooling system. This stability could again be supported by the temperature profile, as can be seen in Fig. 5c. For this purpose, a constant current of 200 A was maintained for approximately 2 min prior to discharge. During the entire current charging and discharging process, the coil temperatures remained constant. These results show that the stability of the MgB_2_ coil under cooling was greatly enhanced in the SN_2_ environment compared to pure conduction cooling. This work is the first to show such a stable high current operation in any MgB_2_ coil in a SN_2_ environment above 25 K, which is very promising for the development of advanced technology for low-cost MRI^43^.

During cool-down, the coil was charged with the full 200 A current several times to determine if there was any effect due to SN_2_ contraction on the coil performance^26^, and no variation in performance was observed. It is worthwhile to note that the coil was not impregnated to avoid conductor movement while the coil was charging. The SN_2_ acted well in place of epoxy and provided very good mechanical and thermal stability to the coil. After cooling down to 8 K, the coil temperature was controlled at 28 K, and again, the coil was able to carry a 200 A current without any performance degradation during thermal cycling. Finally, at the coil temperature of ~28 K, the cryocooler was turned-off to see the warm-up characteristics of the coil. This would be strong evidence that a superconducting magnet with solid cryogen has the potential to operate without system damage under unstable and/or unreliable electrical supply conditions.

Figure 5d shows the temperature versus time plots for the cooling system for 100 h after switching-off the cryocooler. As can be seen in the Figure, the temperature of the radiation shield (only conduction cooling) started to increase rapidly, because it was directly connected to and only cooled by the cryocooler. On the other hand, the temperature of the SN_2_ chamber increased very slowly, even though the cryocooler was also connected to the SN_2_ chamber. As with cooling-down, the warming-up of the SN_2_ around the coil was quite uniform. Figure 5e shows the temperature versus time plot of the coil up to 35 K, which is the *T*_c_ of the coil. Soon after turning-off the cryocooler, the temperature on the coil decreased for some time to achieve temperature equilibrium with the other temperatures in the SN_2_ chamber. The temperature variation occurred during the temperature control process. As shown in Fig. 5e, once temperature equilibrium was achieved, the temperatures on the coil increased slowly and uniformly compared to the pure conduction cooling system. Impressively, it took about 21 h to reach the *T*_c_ of the coil, even though the cryocooler was delivering significant conduction heat load to the SN_2_ chamber. The warming-up time can be greatly increased by thermally disconnecting the cryocooler from the SN_2_ chamber once it is turned-off. Therefore, this system can offer long maintenance-free periods or less re-cooling time in the event of a problem or power failure in the commercial MRI system.

## Conclusions

We designed and developed a SN_2_ cooling system and tested an MgB_2_ solenoid coil at around 30 K. For this purpose, various FEA simulations were first carried out to refine the thermal and mechanical designs of the system. Secondly, the use of a Cu flange was considered to minimize the temperature gradient inside the SN_2_ chamber. As a result, the SN_2_ chamber designed in this study was successfully cooled down to 8 K in combination with a GM cryocooler. In this system, tests on the MgB_2_ solenoid coil were subsequently carried out, such as for *T*_c_ (35 K) and *I*_c_ (>200 A). The MgB_2_ solenoid coil showed very stable current-carrying capacity of 200 A, even at 28 K, without any marked temperature increase. Furthermore, upon turning-off the cryocooler at ~28 K, it took about 21 h for the coil to reach 35 K (the *T*_c_ of the coil) in SN_2_. Such a slow warming-up of the SN_2_ environment can offer long maintenance-free time, less re-cooling time, or even cooling-source-free operation of an MRI magnet in the event of power failure.

## Additional Information

**How to cite this article**: Patel, D. *et al*. Solid cryogen: a cooling system for future MgB_2_ MRI magnet. *Sci. Rep.* **7**, 43444; doi: 10.1038/srep43444 (2017).

**Publisher's note:** Springer Nature remains neutral with regard to jurisdictional claims in published maps and institutional affiliations.

## Supplementary Material

Supporting Information

## Figures and Tables

**Table 1:** Summary of the estimated heat loads on the SN_2_ cooling system.

Thermal heat load	Radiation shield (W)	SN_2_ chamber (W)
Conduction	15.64	0.37
Current lead joule heating at 200 A	26.49	0.26
Radiation (10 layered MLI was used)	2.63	0.01
Residual gas conduction	0.34	0.08
Total (W) (without current)	18.61	0.46
Total (W) (with 200 A current)	45.10	0.72

**Table 2:** Summary of the mechanical design parameters and FEA simulation results.

Mechanical design parameters	SN_2_ chamber	Radiation shield	Cryostat
Design pressure (MPa)	0.30 (internal)	—	0.30 (internal)
Allowable pressure (MPa)	0.30 (internal)	—	0.45 (internal), 1.07 (external)
Calculated cylinder thickness (mm)	1.70	—	—
Cylinder thickness (mm)	3.40	2.00	4.78
Calculated end flange thickness (mm)	7.84	—	14.62
End flange thickness (mm)	8.00	3.00	15.00
FEA simulated von Mises stress (MPa)	9.17	16.31	91.68
FEA simulated total displacement (mm)	0.004	0.28	0.20

**Table 3:** The specifications of the MgB_2_ solenoid coil.

Parameters	Specifications
Coil type	Solenoid
Winding method	Wind and react
Strand (HTR 3520S)	MgB_2_/Nb/Cu/Monel Nb: barrier, Cu: matrix, Monel: sheath
Filament count	36 + 1 (Cu at centre)
Insulation	S-glass
Wire diameter with insulation (mm)	1.3
Wire diameter without insulation (mm)	1.1
SC fill factor of the wire (%)	11.1
Coil I.D./O.D./height (mm)	130/135.2/15
Total turns	23 (1^st^:11, 2^nd^:12)
Total layers	2
Coil filling factor (%)	56
Impregnation	No
Inductance, *L* (μH)	123 (calculated from FEA simulation)
	128 (calculated from inductive voltages)
Field constant (G · A^−1^)	2.19 (calculated from FEA simulation)
	2.22 (measured using Hall sensor)
