# Supplementary material for: Solid cryogen: a cooling system for future MgB2 MRI magnet
Source: Sci Rep. 2017 Mar 2;7:43444. doi: 10.1038/srep43444 (PMC5333112; doi:10.1038/srep43444)
Supplement: Supporting Information [file srep43444-s1.pdf]

Supporting Information

Solid cryogen: a cooling system for future MgB_2 MRI magnet

Dipak Patel[a], Md Shahriar Al Hossain[a*], Wenbin Qiu[a], Hyunseock Jie[a], Yusuke Yamauchi[a], Minoru Maeda[b], Mike Tomsic[c], Seyong Choi[d*], and Jung Ho Kim[a*]

[a]Institute for Superconducting and Electronic Materials, Australian Institute for Innovative Materials, University of Wollongong, Squires Way, Innovation Campus, North Wollongong, New South Wales 2500, Australia

[b]Department of Physics, College of Science and Technology, Nihon University, Tokyo 101-8308, Japan

[c]Hyper Tech Research, Inc., 539 Industrial Mile Road, Columbus, Oh 43228, USA

[d]Busan Center, Korea Basic Science Institute, Busan 609-735, Republic of Korea

[*]Corresponding Authors' email: jhk@uow.edu.au, sychoi07@kbsi.re.kr, shahriar@uow.edu.au

Estimation of Various Components of Total Heat Load

For estimating the total thermal heat load on the solid nitrogen (SN_2) chamber and the radiation shield, the conduction, radiation, and residual gas conduction heat loads were taken into account. The radiation transmission and the gaseous conduction through the access tubes were neglected as in this type of the cooling system, they are approximately 20 and 10 times lower than the conduction heat load of the access tubes, respectively[1].

The conduction heat load (Q) was estimated using equation (1).

$$Q = \frac{A}{L}\int_{T_L}^{T_H} K\, dT \qquad (1)$$

where A, L, T_H, T_L, and K are the cross-sectional area, length, high temperature, low temperature, and thermal conductivity function of the conducting object, respectively.

The conduction heat load via each access tube, current lead and instrumentation wire was individually calculated. To estimate the total conduction heat load, all individual conduction heat loads were added together. Several finite element analysis simulations were carried out for the estimation of temperatures at the various locations in the cooling system while conducting heat load estimation.

The radiation heat load (Q_{rad}) was estimated by Stefan-Boltzmann law using equation (2).

$$Q_{rad} = \varepsilon \cdot \sigma \cdot \left({T_H}^4 - {T_L}^4\right) \cdot A \tag{2}$$

where ε, σ, T_H, T_L, and A are the total effective emissivity, Stefan-Boltzmann constant, temperature of the radiation source, temperature of the radiation receiver, and area of the radiation receiver, respectively.

The radiation configuration of this system resembles parallel flanges, so the total effective emissivity was calculated using equation (3)[2].

$$\varepsilon = \frac{\varepsilon_H \cdot \varepsilon_L}{\varepsilon_H + \varepsilon_L - \varepsilon_H \varepsilon_L} \tag{3}$$

where ε_H, and ε_L are the emissivity of the radiation source, and receiver, respectively.

The radiation heat load was minimized by wrapping multilayer insulation (MLI) around the SN_2 chamber and radiation shield. In the presence of the MLI layers, the equation (2) could be modified to equation (4). This equation is only valid when a hot body is not touching an MLI. If a hot body touches an MLI, the conduction heat load through the MLI must be taken into account.

$$Q_{rad} = \frac{\varepsilon}{N+1} \cdot \sigma \cdot \left({T_H}^4 - {T_L}^4\right) \cdot A \tag{4}$$

where N is the number of MLI layers (i.e. 10 in this work).

For estimating the residual gas conduction (Q_{rgc}), equation (5) can be used[2,3], although, the empirical values given in references[2,3] were used for the residual gas conduction calculations for the radiation shield and the SN_2 chamber.

$$Q_{rgc} = \eta_{rgc} P_{rgc} (T_H - T_L) \tag{5}$$

where η_{rgc} is dependent on high and low temperatures but also depend on the accommodation coefficient[2], and P_{rgc} is the pressure inside the system (i.e. vacuum).

The joule heating (Q) was calculated using Joules' law as per equation (6).

$$Q = I^2 \cdot R \tag{6}$$

where I and R are current and resistance of the conductor, respectively.

References

1 Haid, B. J. *et al.* Design analysis of a solid nitrogen cooled "permanent" high-temperature superconducting magnet system. *Cryogenics* **42**, 617-634 (2002).

2 Iwasa, Y. *Case studies in superconducting magnets, design and operation issues*. 2nd ed., 107-108 (Springer, 2009).

3 Takashi Noguchi. Vacuum insulation for a cryostat. *Teion Kogaku (J. Cryo. Supercond. Soc. Jpn.)* **28,** 355-365 (1993).
